# The industry impact of the American Medical Association’s Digital Medicine Payment Advisory Group (DMPAG)

**DOI:** 10.1038/s41746-022-00743-1

**Published:** 2022-12-24

**Authors:** Joseph C. Kvedar, Mirja Mittermaier, Jordan Pritzker

**Affiliations:** 1 Harvard Medical School, Boston, MA USA; 2 Charité – Universitätsmedizin Berlin, Corporate Member of Freie Universität Berlin and Humboldt-Universität zu Berlin, Department of Infectious Diseases and Respiratory Medicine, Charitéplatz 1, 10117 Berlin, Germany; 3 Berlin Institute of Health at Charité—Universitätsmedizin Berlin, Charitéplatz 1, 10117 Berlin, Germany; 4 CVS/Aetna Health, Inc., Clermont, FL USA

**Keywords:** Business, Health policy

## Abstract

Digital medicine interventions are currently transforming health care and have created new efficiencies in the delivery process. The business model along with physician payment models are crucial drivers for the adoption of innovations. In the U.S., physician payment is mostly codified in the Current Procedural Terminology (CPT). Until recently, CPT codes related to digital medicine activities were mainly limited to telephone services. To embrace the evolving implementation of the various modalities of digital medicine, the American Medical Association (AMA) determined that a more comprehensive codeset is needed. Thus, the Digital Medicine Payment Advisory Group (DMPAG) was initiated in late 2016. Since then, the DMPAG has achieved a significant and measurable impact on digital medicine intervention adoption by introducing CPT codes for remote physiologic monitoring, remote therapeutic monitoring, artificial intelligence, and other digital innovations.

Digital medicine interventions (e.g., telehealth, digital therapeutics, and artificial intelligence) have opened new channels for health care delivery and created new efficiencies in the health care delivery process^1^. This contrasts with other innovations such as novel therapeutics, diagnostics, and devices that also enhance the care delivery process. Digital medicine interventions have faced more adoption challenges than those less disruptive to the care delivery process^2,3^. A critical driver of the adoption of innovations in care delivery is the business model^4^. The majority of healthcare decisions involve physicians, and accordingly, physician payment models are an essential lever for adoption of digital medicine interventions^5^. In the U.S., virtually all physician payment is codified in a set of codes referred to as Current Procedural Terminology (CPT, “www.ama-assn.org/amaone/cpt-current-procedural-terminology”). CPT is governed by the American Medical Association (AMA, “www.ama-assn.org”). The CPT code set codifies the level of physician work and/or practice expense related to a care delivery process. Code Change Applications (CCA) for new codes or updates to existing codes are presented to the CPT Editorial Panel three times per year. If the panelists feel that there is sufficient evidence to support the code’s use and that the CCA is not represented by the current code set, the panelists will approve the CCA. The approved CCA then goes to the RUC (RBRVS (Resource-Based Relative Value Scale) Utilization Committee) for suggested valuation which is then submitted to the U.S. Centers for Medicare and Medicaid Services (CMS, “www.cms.gov”). CMS determines if Medicare will pay for the CPT code. Many private (commercial) insurers consider the CMS coverage and valuation decisions when making their coverage and payment decisions for health care provider services represented by CPT codes. Many of the CCAs related to digital medicine intervention have been submitted to the AMA CPT Panel by the Digital Medicine Payment Advisory Group (DMPAG, “www.ama-assn.org/practice-management/digital/digital-medicine-payment-advisory-group”).

Until recently, physician payment for digital medicine activities was scarce and mainly limited to telehealth consultations for patients in Health Profession Shortage Areas as defined by CMS. Digital therapeutics, remote physiologic monitoring, artificial intelligence, and other digital innovations reached the marketplace during this phase.

In 2015, the CPT Editorial panel assembled the Telehealth Services Workgroup to address the growth of interest in telehealth. The initial work product of this group was Appendix P (CPT Codes That May Be Used for Synchronous Telemedicine Services) of the CPT code set. Though this was viewed as a positive step, the AMA leadership determined that a more comprehensive view was needed. In late 2016, the multi-stakeholder DMPAG was convened. The DMPAG consists of 18 people, 50% digital medicine experts or practitioners and 50% experts in the CPT process.

The achievement record of the DMPAG has been remarkable. At the time of inception, there was no reimbursement path for digital medical interventions such as remote physiologic monitoring, remote therapeutic monitoring, asynchronous exchange of information between doctor and patient, online consultation between a primary provider and a specialist, or use of any healthcare-related mobile applications.

Over the first six years of the DMPAG, the group’s impact on digital medicine adoption has been significant and measurable. CPT codes for services in the paragraph above now exist and are paid by CMS and many private insurers. Table 1 lists a compilation of these services and their corresponding codes published in the CPT manual.

**Table 1:** CPT codes and appendices developed by the DMPAG.

CPT code	CPT code numbers	Effective date
Remote Physiologic Monitoring	99453, 99454, 99457, 99458	January 1, 2019 (99458 effective date January 1, 2020)
Interprofessional telephone/internet/EHR consultation (eConsults)	99451, 99452	January 1, 2019
Online Digital Evaluation and Management (eVisit)	99421, 99422, 99423, 98970, 98971, 98972	January 1, 2020
Remote Therapeutic Monitoring	98975, 98976, 98977, 98980, 98981	January 1, 2022
Digital Medicine-Services Taxonomy	Appendix R	January 1, 2022
AI taxonomy for medical services & procedures	Appendix S	January 1, 2022

When new reimbursement codes are introduced and adopted by CMS, it takes several years to see measurable reimbursement activity. The earliest codes to be promulgated by the DMPAG were codes to support the care of patients using remote physiologic monitoring (RPM) technologies. These codes were conceived in 2017 and became part of the CMS-reimbursed code set in 2019. We have two years of data on their use in Medicare, and as illustrated in Fig. 1, the growth in the utilization of these codes was substantial. Codes for chronic care management (CCM), released in 2016, are offered for comparison. The growth trajectory is substantially less. Quite likely, the COVID-19 pandemic played an important role in catalyzing RPM code reimbursement adoption. This further illustrates that the DMPAG has kept pace with the implementation of digital medicine interventions.

**Fig. 1:**
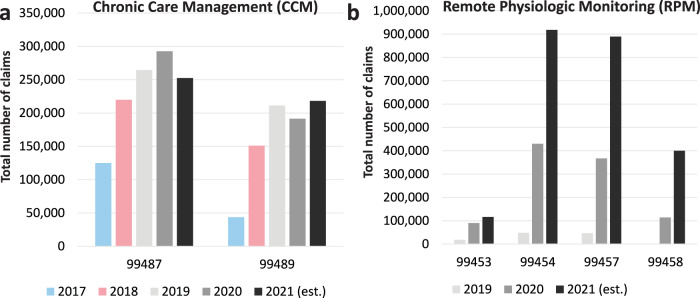
CPT codes of Chronic Care Management (CCM) and Remote Physiologic Monitoring (RPM). **a** The CCM codes stand for: 99487: CCM services, with the following required elements: multiple (two or more) chronic conditions expected to last at least 12 months, or until the death of the patient; first 60 min of clinical staff time directed by a physician or other qualified health care professional, per calendar month; 99489: add-on code: each additional 30 min of clinical staff time directed by a physician or other qualified health care professional, per calendar month. **b** The RPM codes are defined as: 99453: RPM parameter(s) (e.g., weight, blood pressure, pulse oximetry, respiratory flow rate), initial; set-up and patient education on use of equipment; 99454: Device(s) supply with daily recording(s) or programmed alert(s) transmission, each 30 days; 99457: RPM treatment management services, 20 min or more of clinical staff/physician/other qualified healthcare professional time in a calendar month requiring interactive communication with the patient/caregiver during the month; 99458: RPM treatment management services, clinical staff/physician/other qualified health care professional time in a calendar month requiring interactive communication with the patient/caregiver during the month; additional 20 min.

Following the RPM code implementation, the DMPAG was instrumental in developing and refining the parallel set of codes for the gap in CPT coding for remote therapeutic monitoring (RTM) technologies. These RTM codes are new enough that, to date, the utilization data for the RTM codes is limited.

Another example of a code born of the DMPAG’s efforts allows for reimbursement for so-called e-consults, where a clinician (often a primary care clinician) sends relevant information about a complex case to a specialist. The specialist can now be reimbursed for that activity. Fig. 2 illustrates the growth in the utilization of those codes.

**Fig. 2:**
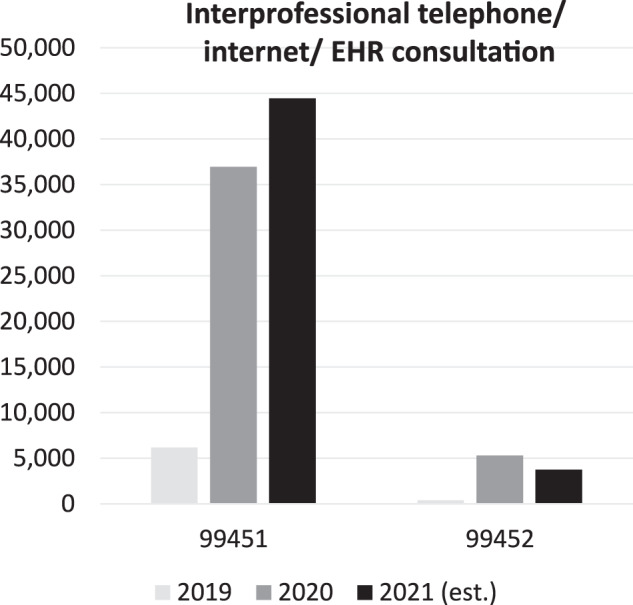
CPT codes of Interprofessional telephone/ internet/ EHR consultation. The CPT codes are defined as: 99451: Interprofessional telephone/internet/electronic health record assessment and management service provided by a consultative physician, including a written report to the patient’s treating/requesting physician or other qualified health care professional, 5 min or more of medical consultative time; 99452: Interprofessional telephone/internet/electronic health record referral service(s) provided by a treating/requesting physician or other qualified health care professional, 30 min.

Since DMPAG’s initiation in 2016, digital therapeutics, artificial intelligence (sometimes referred to as augmented intelligence), and other digital innovations have reached the marketplace and the AMA has attempted to resolve the complexity of appropriate reimbursement for digital medicine. The DMPAG has taken its moniker of ‘digital medicine’ seriously and not focused simply on telehealth and remote care. A recent example of another foray into technology-related reimbursement has been efforts to create a taxonomy for using augmented intelligence in healthcare delivery (Table 2).

**Table 2:** Taxonomy for the use of augmented intelligence in healthcare.

Service components	AI category: assistive	AI category: augmentative	AI category: autonomous
Primary objective	Detects clinically relevant data	Analyzes and/or quantifies data in a clinically meaningful way	Interprets data and independently generates clinically meaningful conclusions
Provides independent diagnosis and/or management decision	No	No	Yes
Analyzes data	No	Yes	Yes
Requires physician or other qualified health care professional interpretation and report	Yes	Yes	No
Examples in CPT code set	Computer-Aided Detection (CAD) Imaging (77048, 77049, 77065-77067, 0042 T, 0174 T, 0175 T)	Magnetic Resonance Spectroscopy (0612 T), external analysis of imaging data sets	Retinal Imaging (92229)

What makes the DMPAG successful? The group formed a productive culture and chemistry early on. The AMA has devoted tremendous resources to giving the group a perspective on upcoming challenges in digital medicine payment and the tools needed to gather the data required to make thoughtful policy recommendations.

What is in the future for the DMPAG? The AMA has expressed enthusiasm to continue convening the group. While many of the goals we set out initially have been achieved, we’ve come upon a phase of challenging work that will continue into the coming years. This work is partially opportunistic. The DMPAG is forming several partnerships with adjacent digital medicine advocacy groups and regularly hearing from digital medicine commercial entities who recognize coding gaps as they scale their business models. The other part of the work of the DMPAG is deliberate and involves a foray into understanding how to reimburse for a series of technologies that are intended to improve quality outcomes and safety of patient care while increasing physician efficiency. CPT code values are calculated based on three dimensions: the complexity of medical decision-making, time spent, and practice expense. Tools such as augmented intelligence and digital therapeutics improve some aspects of care delivery by automating work previously done by people. As we determine how to value these services as they are integrated into care delivery, we are working to resolve how to facilitate payment mechanisms for providers when they do less work or, conversely, the increased provider work as a result of more information available to consider for the care of the patient.

## Data Availability

All data necessary to support the results are provided in this article. The data are available from the corresponding author upon reasonable request.
